# Investigation of adenosine A1 receptor-mediated β-arrestin 2 recruitment using a split-luciferase assay

**DOI:** 10.3389/fphar.2023.1172551

**Published:** 2023-05-30

**Authors:** Luisa Saecker, Hanns Häberlein, Sebastian Franken

**Affiliations:** Institute of Biochemistry and Molecular Biology, Medical Faculty, University of Bonn, Bonn, Germany

**Keywords:** adenosine A1 receptor, β-arrestin 2, cAMP, nanoluciferase, live cell assay, valerian

## Abstract

**Background:** Adenosine A1 receptor (A_1_AR) plays a prominent role in neurological and cardiac diseases and inflammatory processes. Its endogenous ligand adenosine is known to be one of the key players in the sleep–wake cycle. Like other G protein-coupled receptors (GPCRs), stimulation of A_1_AR leads to the recruitment of arrestins in addition to the activation of G proteins. So far, little is known about the role of these proteins in signal transduction and regulation of A_1_AR compared to the activation of G proteins. In this work, we characterized a live cell assay for A_1_AR-mediated β-arrestin 2 recruitment. We have applied this assay to a set of different compounds that interact with this receptor.

**Methods:** Based on NanoBit^®^ technology, a protein complementation assay was developed in which the A_1_AR is coupled to the large part of the nanoluciferase (LgBiT), whereas its small part (SmBiT) is fused to the N-terminus of β-arrestin 2. Stimulation of A_1_AR results in the recruitment of β-arrestin 2 and subsequent complementation of a functional nanoluciferase. For comparison, corresponding data on the effect of receptor stimulation on intracellular cAMP levels were collected for some data sets using the GloSensor™ assay.

**Results:** The assay gives highly reproducible results with a very good signal-to-noise ratio. Capadenoson, in contrast to adenosine, CPA, or NECA, shows only partial agonism in this assay with respect to the recruitment of β-arrestin 2, whereas it shows full agonism in the case of the inhibitory effect of A_1_AR on cAMP production. By using a GRK2 inhibitor, it becomes clear that the recruitment is at least partially dependent on the phosphorylation of the receptor by this kinase. Interestingly, this was also the first time that we demonstrate the A_1_AR-mediated recruitment of β-arrestin 2 by stimulation with a valerian extract.

**Conclusion:** The presented assay is a useful tool for the quantitative study of A_1_AR-mediated β-arrestin 2 recruitment. It allows data collection for stimulatory, inhibitory, and modulatory substances and is also suitable for more complex substance mixtures such as valerian extract.

## Introduction

The ubiquitous endogenous molecule adenosine is well-studied and known to be part of nearly all cellular processes. It arises primarily from the breakdown of adenosine triphosphate (ATP), which is one of the major metabolites in living organisms (Sheth et al., 2014). Adenosine interacts with adenosine receptors (ARs) that belong to the superfamily of G protein-coupled receptors (GPCRs). ARs are divided into four subtypes, namely, A_1_AR, A_2A_AR, A_2B_AR, and A_3_AR. They are involved in many different physiological and pathological processes and gained high interest as pharmaceutical targets (Sheth et al., 2014; Borea et al., 2018; Pasquini et al., 2022). ARs are expressed in several different cells, tissues, and major organs, including the brain, lungs, heart, liver, and kidney. A_1_AR, in particular, is highly expressed in the brain and central nervous system (CNS), predominantly in the cortex, hippocampus, cerebellum, spinal cord, and glial cells (Fastbom et al., 1986; Reppert et al., 1991; Dixon et al., 1996). The receptors have different affinities for adenosine: A_1_AR and A_2A_AR have high affinity, and A_2B_AR and A_3_AR have low affinity (Boison, 2008). The binding of an agonist usually leads to a conformational change in the receptor, resulting in the activation of downstream signaling via G proteins consisting of α, β, and γ subunits (Ranjan et al., 2017). A_1_AR and A_3_AR are coupled to G_i/o_ proteins, resulting in the inhibition of adenylate cyclase, while A_2A_AR and A_2B_AR are coupled to G_s/olf_ proteins, leading to the stimulation of adenylate cyclase. Therefore, activation of A_1_AR and A_3_AR inhibits cyclic adenosine monophosphate (cAMP) formation, resulting in decreased protein kinase A (PKA) activity and phosphorylation of cyclic AMP response element-binding protein (CREB). Stimulation of A_2A_AR and A_2B_AR and *vice versa* increase the formation of cAMP, leading to the activation of PKA and phosphorylation of CREB (Sheth et al., 2014). A_1_AR also activates phospholipase C (PLC), leading to an increase in inositol 1,4,5-triphosphate (IP3), resulting in calcium release from the endoplasmic reticulum (ER) into the cytosol (Gerwins and Fredholm, 1992). In addition to G protein-dependent signaling pathways, ARs are also known to induce G protein-independent signaling pathways. Such pathways are initiated by receptor phosphorylation through G protein-coupled receptor kinases (GRKs) and result in the binding of scaffold proteins like β-arrestins. In addition to receptor desensitization, recruitment of β-arrestins can also promote downstream signaling (Klaasse et al., 2008; Shenoy and Lefkowitz, 2011; Rajagopal and Shenoy, 2018). Four different arrestin isoforms are known, 1 and 4 are so-called visual arrestins and 2 and 3 are non-visual. The latter are also referred to as β-arrestin 1 and 2, respectively. The two non-visual isoforms together with the ubiquitously expressed GRKs play a key role in the regulation of GPCR signaling (Shenoy & Lefkowitz, 2011; Gurevich & Gurevich, 2019; Chen & Tesmer, 2022). It is described that stimulated A_1_AR recruits β-arrestins. This can either lead to receptor desensitization (Klaasse et al., 2008) and/or mediates downstream signaling (Schulte and Fredholm, 2003; Verzijl and Ijzerman, 2011). However, there is still very limited data for A_1_AR-mediated β-arrestin recruitment compared to other GPCRs.

To characterize β-arrestin 2 recruitment, different tools based on technologies like Förster resonance energy transfer (FRET), bioluminescence resonance energy transfer (BRET), and Tango or different kinds of protein complementation can be used (Hoare and Hughes, 2004; Hoare et al., 2021; Perry-Hauser et al., 2021; Guo et al., 2022). In this study, a direct cellular luciferase assay using the NanoBit^®^ system was evaluated for its suitability to investigate A_1_AR ligands. The system uses a setup of two fragments that form a functional nanoluciferase when they come in close proximity. One fragment called Large BiT (LgBiT) is fused to the receptor, while the corresponding smaller fragment Small BiT (SmBiT) is fused to β-arrestin 2. Luminescence, as the result of substrate conversion catalyzed by the resulting enzyme, is measured in real-time. The assay system allows quantification of the specific recruitment of β-arrestin 2 initiated by compounds interacting with A_1_AR.

## Materials and methods

### Biochemicals and reagents

HEK 293 cells were obtained from the German Collection of Microorganisms and Cell Cultures GmbH (DSMZ, Braunschweig, Germany; ACC 305). Research reagents and chemicals were received from the following suppliers: Dulbecco’s modified Eagle medium (DMEM; Thermo Fisher Scientific, Waltham, MA, United States; 31,885-023), fetal bovine serum (FBS; Thermo Fisher Scientific, A5256701), trypsin–EDTA 0.05% (Thermo Fisher Scientific, 25300062), penicillin-streptomycin 10.000 U/mL (PenStrep; Thermo Fisher Scientific, 15140122), phosphate-buffered saline (PBS; Thermo Fisher Scientific, 10010023), coelenterazine h (Prolume Ltd., Pinetop, AZ, United States; 50909-86-9), GloSensor™ cAMP Assay Reagent (Promega, E1290), Zeocin™ Selection Reagent (Thermo Fisher Scientific, R25001), Hygromycin B Gold (InvivoGen, San Diego, Ca, United States; ant-hg-1), and Geneticin™ G-418 Sulfate (Thermo Fisher Scientific, 108,321-42-2). A 20 mM HEPES-buffered Hanks balanced salt solution (HBSS/HEPES) was freshly prepared in the laboratory.

The test ligands were obtained from the following suppliers: N6-cyclopentyladenosine (CPA; Cayman Chemicals, Ann Arbor, MI, United States; 41,552-82-3), 5′-N-ethylcarboxamidoadenosine (NECA; Sigma-Aldrich, St. Louis, MO, united States; 35920-39-9), adenosine (Ado; Sigma-Aldrich, 58-61-7), 8-cyclopentyl-1,3-dipropylxanthine (DPCPX; Tocris, Bristol, United Kingdom; 102146-07-6), 4-[2-[[6-amino-9-(N-ethyl-β-D-ribofuranurona mi dosyl)-9H-purin-2-yl]amino] ethyl]-benzenepropanoic acid, monohydrochloride (CGS 21680; Cayman Chemicals, 124431-80-7), 2-[[6-amino-3,5-dicyano-4-[4-(cyclopropylmethoxy) phenyl]-2-pyridinyl]thio]-acetamide (BAY 60-6583; Tocris, 910487-58-0), 1-[2-chloro-6-[[(3-iodophenyl)methyl]amino]-9H-purin-9-yl]-1-deoxy-N-methyl-β-D-ribofuranuronamide (2-Chloro-IB-MECA; Cayman Chemicals, 163042-96-4), [2-Amino-4-[3-(trifluoromethyl)phenyl]-3-thienyl] phenylmethanone (VCP 171; Tocris, 1018830-99-3), 5-[2-(5-nitro-2-furanyl)ethenyl]-2-furancarboxylic acid, methyl ester (βARK1/GRK2 inhibitor; Cayman Chemicals, 24269-96-3), capadenoson (Cayman Chemicals, 544417-40-5), isoprenaline hydrochloride (Iso; Sigma-Aldrich, 51-30-9), and forskolin (FSK; Sigma-Aldrich, 66575-29-9). Valerian extract Ze 911 was kindly provided by Max Zeller Söhne AG (Romanshorn, Switzerland). Ze 911 corresponds to the European Pharmacopoeia monograph *valerian dry hydroalcoholic extract*. The extraction solvent was 50.8% methanol (v/v) leading to a drug–extract ratio of 4–6:1. The characteristic ingredients are sesquiterpenic acids like valerenic acid, hydroxyvalerenic acid, and acetoxyvalerenic acid (Vissiennon et al., 2006). Ze 911 contains a minimum of 0.25% sesquiterpenic acid expressed as valerenic acid.

### Generation of expression plasmids and stably expressing cell lines

The plasmid coding for human A_1_AR fused to the N-terminus of the Large BiT (pCMV_ADORA1-LgBit) was generated by amplifying the coding region of A_1_AR by addition of a HindIII site and a BamHI site to the 5′- and 3′-end, respectively, using PCR (forward primer: 5′-GAT​CAA​GCT​TGA​TAT​GCC​TCC​CAG​TAT​ATC​CG-3’; reverse primer: 5′- GAT​CGG​ATC​CGA​TCG​TCA​GGC​C-GTT​C-3′). The plasmid ADORA1-Tango (Addgene plasmid #66209; http://n2t.net/addgene: 66209; RRID: Addgene_ 66209) used as template was a gift from Bryan Roth (Kroeze et al., 2015). The PCR product was treated with restriction enzymes and ligated via the same sites into a plasmid in front of the sequence for the LgBiT under the control of the CMV promoter.

For the expression of rat β-arrestin 2 with an N-terminal SmBiT, the coding sequence was taken from pECFP-N1_rβ-arrestin-2 (a kind gift from M. Bouvier, Montreal, Canada) by restriction with NheI and SalI. The fragment was introduced into pCDNA™3.1/Zeo (+) Mammalian Expression Vector (Invitrogen) containing the information for the SmBiT via NheI and XhoI sites (pCDNA3 .1Zeo_SmBit-β-arrestin 2).

HEK 293 cells stably expressing A_1_AR-LgBiT and SmBiT-β-arrestin 2 (A_1_AR-NanoBit^®^-βarr2 HEK 293 cells) were produced by double transfection using polyethylenimine (PEI) and selection of positives clones by addition of G418 (700 μg/mL) and zeocin (100 μg/mL).

Cells used for cAMP experiments were produced by PEI transfection of HEK 293 cells with the commercially available plasmid pGloSensor™-22F cAMP (Promega, GU174434). Those cells were additionally PEI-transfected with a plasmid containing the information for the adenosine A1 receptor under the control of a CMV promoter. The cells were selected using G418 (700 μg/mL) and Hygromycin B Gold (100 μg/mL; selection antibiotic of the GloSensor™ system).

Stably transfected cells were cultured in low glucose DMEM supplemented with 10% (v/v) FBS, 100 IU/mL penicillin, and 100 μg/mL streptomycin (PenStrep). The cells were incubated at 37°C and 5% (v/v) CO_2_. The cells were passaged every 3 days at a ratio of 1:10 at a confluency of 80%–90%.

## Establishment of cell-based assay systems

### β-arrestin 2 recruitment assay

The assay was constructed to detect and monitor real-time protein–protein interactions. Once the two fragments LgBiT and SmBiT come in close proximity after receptor activation and phosphorylation, they build a functional enzyme that generates light upon the addition of its substrate fumerazine or coelenterazine (Wouters et al., 2018).

A_1_AR-NanoBit^®^-β arr2 HEK 293 cells were seeded in a white clear bottom 96-well plate (25000 cells/well) and incubated at 37°C and 5% CO_2_ for 24 h. Test compounds were either dissolved in DMSO or in water and then further diluted in water; however, the maximum concentration of DMSO on cells was 0.1%. The medium was replaced by 45 µL coelenterazine h substrate solution (2.5 µM coelenterazine h in HEPES-buffered Hanks balanced salt solution). The 96-well plate was immediately placed into the Tecan Spark^®^ multimode microplate reader, where the background luminescence was measured for three to five cycles until a stable signal was obtained. The measurement was paused, and cells were stimulated with 5 µL ligand solutions. The measurement was continued for another 55–57 cycles.

### GloSensor™ cAMP assay

The establishment of HEK 293 cells expressing a cAMP biosensor and measurement of cAMP were performed as described by Bussmann et al. (2020). The cells were seeded in a white clear bottom 96-well plate (35.000 cells/well) and incubated at 37°C and 5% CO_2_ for 24 h. The medium was changed to 25 µL substrate solution per well containing 4% GloSensor™ cAMP Reagent stock solution in HEPES-buffered DMEM. The cells were incubated for 1 hour at 37°C and subsequently equilibrated for another hour at room temperature in the plate reader (Tecan Spark^®^). The cells in which adenylate cyclase had been activated with forskolin and isoprenaline were stimulated with different A_1_AR agonists. The decrease in cAMP concentration due to A_1_AR stimulation was measured by luminescence differences.

### Data analysis and statistics

In the case of the NanoBit^®^ assay, raw data from three to four independent experiments were collected and transferred to Prism (GraphPad Software, Inc., San Diego, CA, United States, version 9.5.0) and plotted as a function of time. To normalize for well-to-well variabilities, each data point was divided by the mean of the first three values using the “remove baseline and column math” function of the software. Next, the solvent control was subtracted using the same functionality of Prism. After absolute signals were corrected for solvent control samples and inter-well variability, areas under the curve (AUCs) were calculated using the corresponding function in Prism. Mean AUC values from three to four experiments were plotted against the concentration of the agonist used (log M). A sigmoidal curve was fitted to calculate EC_50_ and IC_50_ values based on the dose–response data using a non-linear regression model (variable slope; see also Supplementary Figure S1).

Initial rates were calculated using the plug-in equations provided by Dr. Samuel Hoare (Hoare et al., 2020a). Specifically, the rise-and-fall equation that considers baseline and drift was used to calculate initial rates.

For the GloSensor™ cAMP assay, raw data of single experiments were transferred to Prism and plotted as a function of time. IC_50_ values based on the dose–response data were calculated as described previously. All experiments were repeated at least twice to verify the results.

To detect statistical differences between groups, analysis of variance (ANOVA) followed by *post-hoc* analysis (Dunnett´s) was performed.

## Results

### Different A_1_AR agonists show different efficiencies for β-arrestin 2 recruitment

**FIGURE 1:**
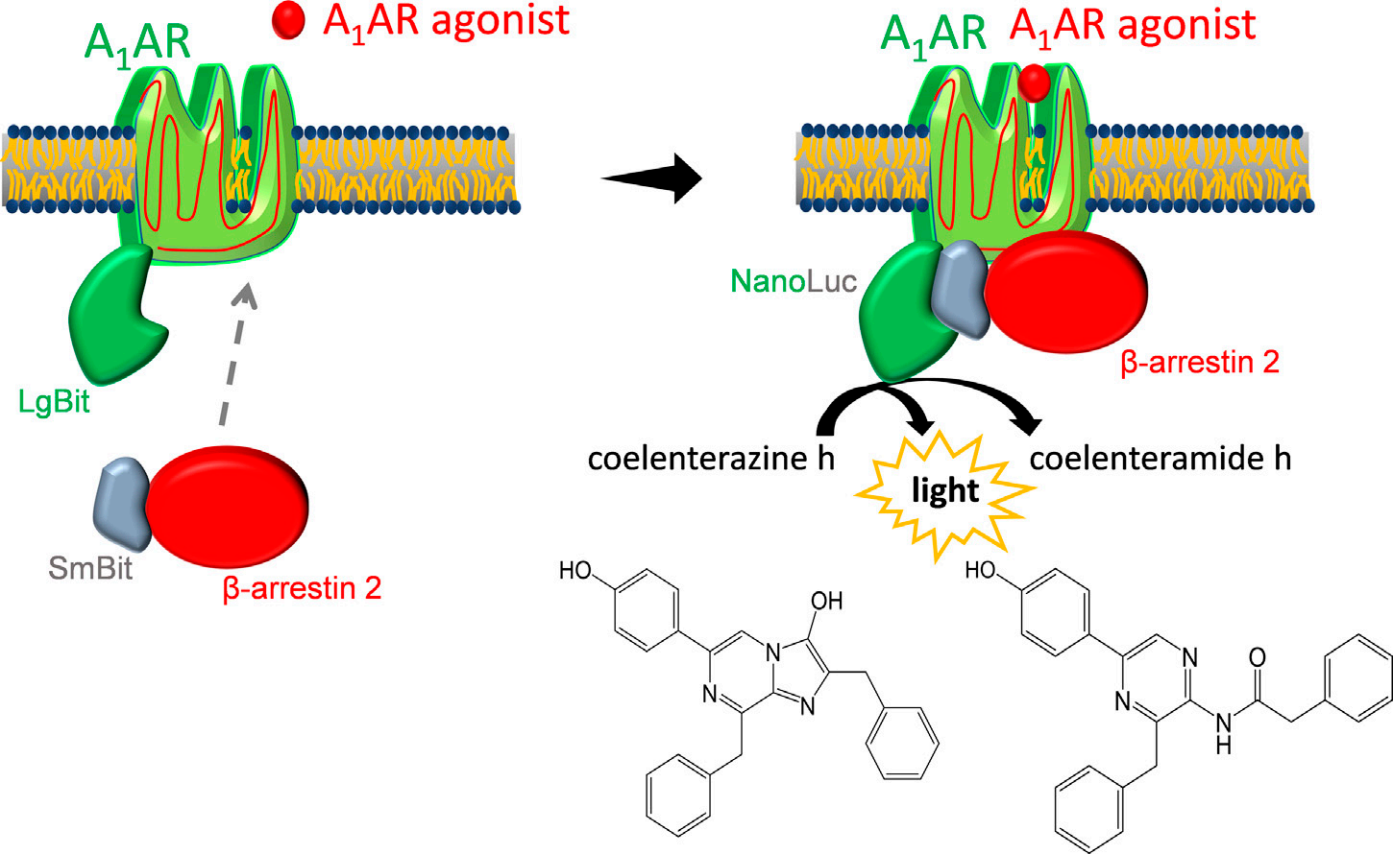
Luciferase-based β-arrestin 2 recruitment assay. After agonist binding, SmBiT-β-arrestin 2 migrates to the adenosine A1 receptor (A_1_AR) that is coupled to LgBiT. Binding of LgBiT and SmBiT results in a functional enzyme (NanoLuc) that generates light in the presence of the substrate coelenterazine h.

**FIGURE 2:**
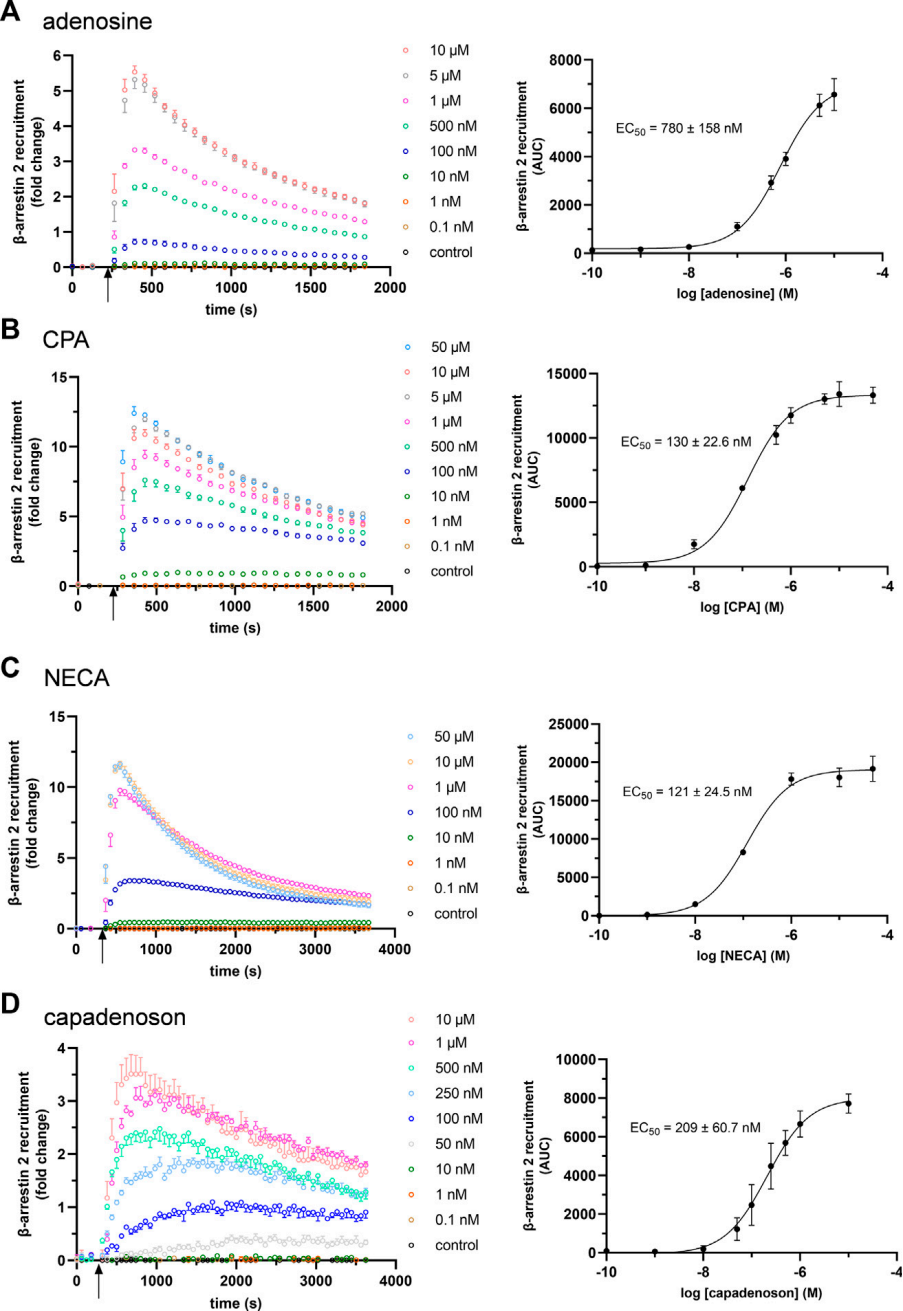
Concentration dependency of β-arrestin 2 recruitment in the A1AR NanoBit® reporter assay: At the time point indicated by the arrow, **(A)** adenosine, **(B)** CPA, **(C)** NECA, and **(D)** capadenoson were added to A1AR-NanoBit®-βarr2 HEK 293 cells at concentrations indicated beside the graph, and luminescence was measured for up to 60 min. A solvent control of 0.1% DMSO was included. Graphs on the left are exemplary for one of three experiments performed in triplicate (see also Supplementary Figure S2 for repeated experiments). Dose–response curves on the right were calculated from all three experiments using areas under the curves (AUCs). Values are given as mean ± SEM (n = 3 independent experiments performed in triplicate).

The established cell-based NanoBit^®^ assay was designed to study β-arrestin 2 recruitment by adenosine A_1_AR in real time. For this purpose, the LgBiT was linked to the C-terminus of the receptor and the SmBiT to the N-terminus of β-arrestin 2 (Figure 1). Similar to what was seen for other ARs (Storme et al., 2018a), this combination resulted in a dose-dependent signal increase after the application of adenosine to transfected HEK 293 cells (Figure 2A; Supplementary Figure S2). Using these data sets, an EC_50_ for adenosine of 780 ± 158 nM was calculated. Furthermore, dose–response experiments for CPA (Figure 2B; Supplementary Figure S2) and NECA were performed (Figure 2C; Supplementary Figure S2). As seen for adenosine, very robust dose–response curves for both agonists were calculated from the AUC values. Compared to adenosine, both agonists showed significantly lower EC_50_ values for β-arrestin 2 recruitment: 130 ± 22.6 nM (CPA; *p* = 0.0030) and 121 ± 24.5 nM (NECA; *p* = 0.0027), respectively (one-way analysis of variance, Dunnett’s *post hoc*).

**FIGURE 3:**
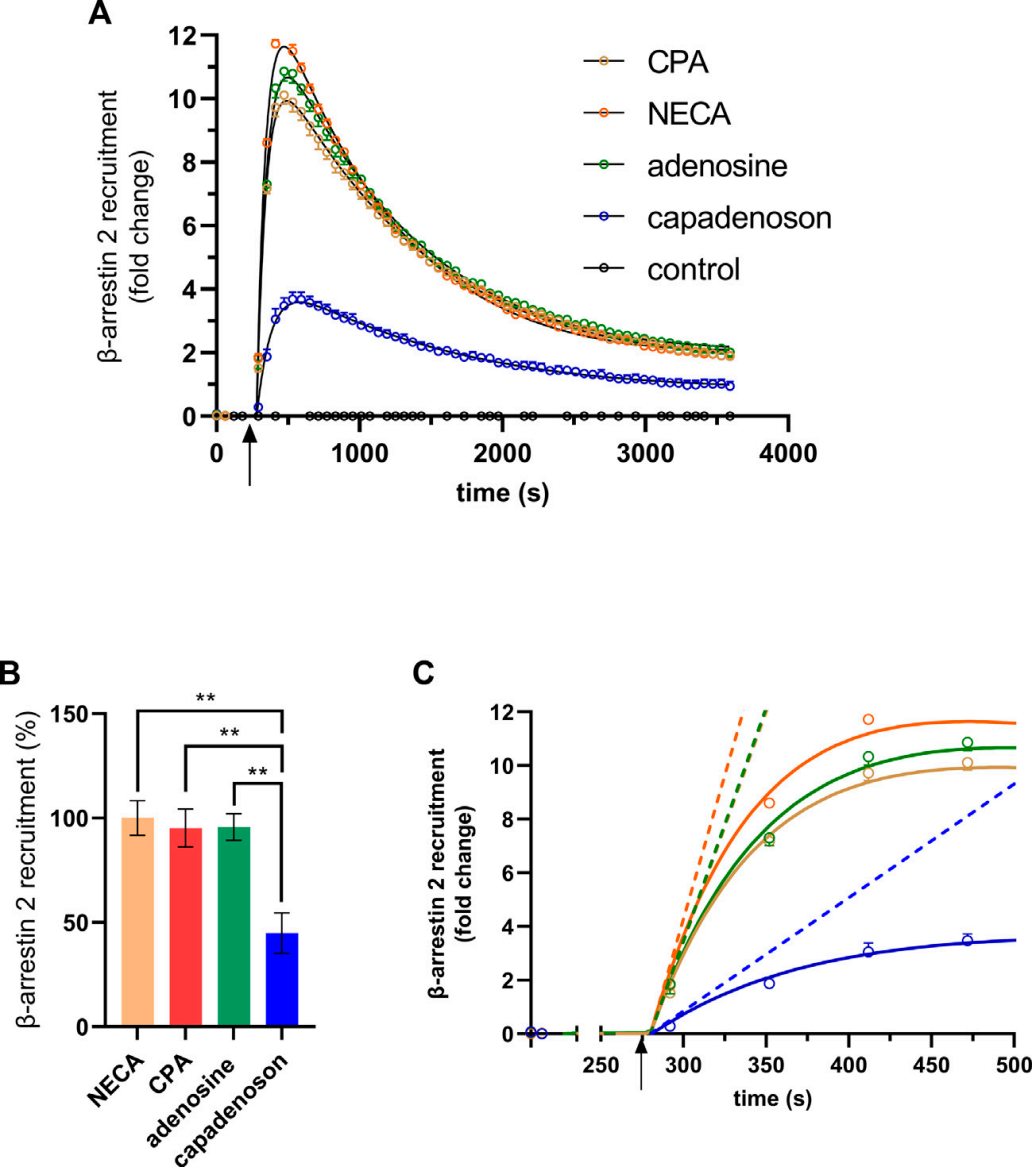
**(A)** β-arrestin 2 recruitment in the A_1_AR NanoBit^®^ reporter assay by different agonists: 10 µM of either CPA, NECA, adenosine, or capadenoson was added to A_1_AR NanoBit^®^-βarr2 HEK 293 cells at the time point indicated by the arrow, and luminescence was measured for 60 min. A solvent control of 0.1% DMSO was included. The graphs are exemplary for one of four experiments performed in triplicate (see Supplementary Figure S3A for repeated experiments). Curves calculated by GraphPad prism plug-in “rise-and-fall equation” provided by Dr. Samuel Hoare are given in black. Values are given as means ± SEM. **(B)** β-arrestin 2 recruitment of different agonists compared to NECA is given in percent. Values are given as means ± SEM (n = 4 independent experiments performed in triplicate). **p < 0.01 value was significantly different (one-way analysis of variance, Dunnet’s *post hoc*) compared to capadenoson. **(C)** Activation phase. Initial rates calculated from the fitted curves shown in **(A)** are given as dashed lines.

Next, we tested the non-nucleoside agonist capadenoson for recruitment of β-arrestin 2. It resulted in dose-dependent recruitment, which was less pronounced than with the other agonists tested (Figure 2D; Supplementary Figure S2). The calculated EC_50_ for capadenoson was 209 ± 60.7 nM. When tested side by side in a saturated concentration, fold change in the luminescent signal was up to three times higher for adenosine, CPA, and NECA than for capadenoson (Figure 3A; Supplementary Figure S3). Calculated by AUC values, capadenoson was about half as efficient as the other agonists tested (Figure 3B).

Calculation of the AUC contains activation as well as deactivation/internalization events of the receptor. In some cases, it can be useful to compare the activation of the receptor only. Hoare et al. developed a tool for evaluating G protein- and arrestin-mediated signaling in living cells, which allows the calculation of receptor activation and deactivation separately (Hoare et al., 2021). This tool was developed using fluorescent reporters, but curves calculated by it fitted quite well to our luminescence-based data. Black lines in Figure 3A were calculated from the fit for the different agonists used in this study. The analysis tool provides different parameters, such as the initial rate. This is the slope of a straight line adapted to the activation phase (Figure 3C). The initial rates calculated from the fits were 10.422 ±± 0.297-fold change/min for adenosine, 10.296 ± 0.265-fold change/min for CPA, and 12.858 ± 0.277-fold change/min for NECA, but only 2.540 ± 0.148-fold change/min for capadenoson.

### Specificity of A_1_AR NanoBit^®^ reporter assay for β-arrestin 2 recruitment to A_1_AR

**FIGURE 4:**
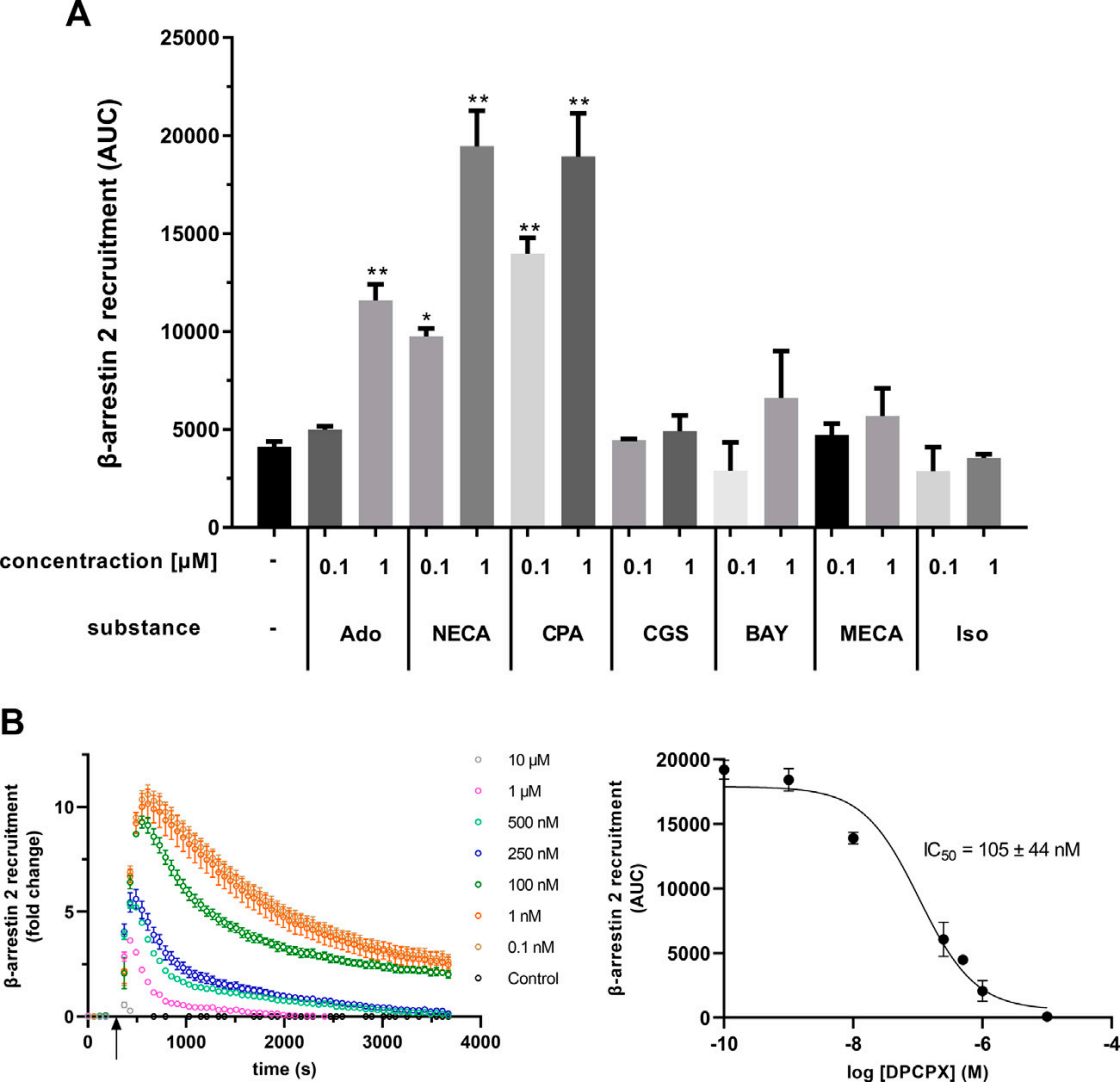
**(A)** Different adenosine receptor agonists were tested in the A_1_AR NanoBit^®^ reporter assay in concentrations of 0.1 and 1 μM, respectively. Adenosine (Ado; unspecific, endogenous agonist), NECA (unspecific agonist), and CPA (specific A_1_AR agonist) led to a significant increase in β-arrestin 2 recruitment, whereas CGS 21680 (CGS; specific A_2_AAR agonist), BAY 60-6583 (BAY; specific A_2_BAR agonist), and 2-Cl-IB-MECA (MECA; specific A_3_AR agonist) did not change the recruitment significantly when compared to control (0.1% DMSO). Isoprenaline (Iso; β-adrenergic agonist) was used as unrelated control. Values are given as means ± SEM (n = 3 independent experiments performed in triplicate). *p < 0.05 and **p < 0.01 values were significantly different (one-way analysis of variance, Dunnett’s *post hoc*) compared to DMSO control. **(B)** Influence of the A_1_AR antagonist DPCPX on β-arrestin 2 recruitment in the A_1_AR NanoBit^®^ reporter assay. A_1_AR-NanoBit^®^-βarr2 HEK 293 cells were incubated with increasing amounts of DPCPX. β-arrestin 2 recruitment was induced using 1 µM CPA. The graph on the left is exemplary for one of three experiments. The dose–response curve for DPCPX on the right was calculated from all three experiments using areas under the curves (AUC). Values are given as mean ± SEM (n = 3 independent experiments performed in triplicate).

Furthermore, the specificity of the assay was investigated with the AR agonists NECA (non-specific), adenosine (non-specific), CPA (specific, A_1_AR), CGS 21680 (specific, A_2A_AR), BAY 60-6583 (specific, A_2B_AR), and 2-Cl-IB-MECA (specific, A_3_AR). Untreated or isoprenaline-treated cells served as controls. The agonists were tested at two concentrations, 0.1 µM and 1 µM. As seen before, both non-specific AR agonists NECA and adenosine as well as the A_1_AR-specific agonist CPA caused significant recruitment of β-arrestin 2 (Figure 4A). Treatment with NECA (9748 ± 407 AUC and 19,461 ± 1809 AUC) and CPA (13,968 ± 824 AUC and 18,926 ± 2220 AUC) resulted in higher recruitment than with adenosine (4988 ± 193 AUC and 11,580 ± 833 AUC). In contrast, agonists specific for the other three ARs did not significantly increase β-arrestin 2 recruitment (Figure 4A). To further test the specificity of the assay, the effect of DPCPX, an A_1_AR inhibitor, on recruitment was examined. DPCPX blocked β-arrestin 2 recruitment by CPA in a dose-dependent manner (Figure 4B). The calculated IC_50_ for DPCPX was 105 ± 44 nM.

### Modulation of A_1_AR/β-arrestin 2 interaction measured by NanoBit^®^ reporter assay

**FIGURE 5:**
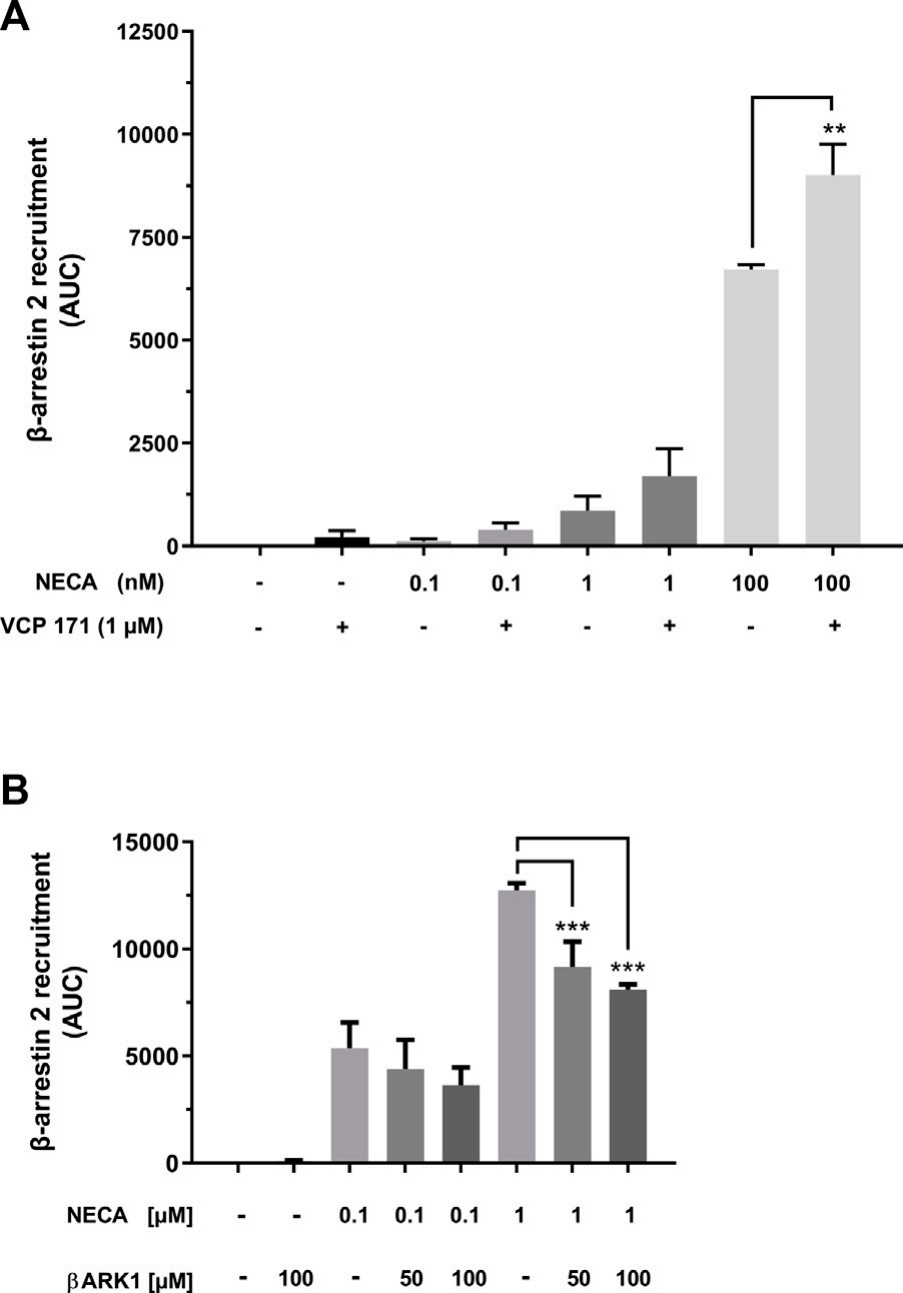
Modulation of β-arrestin 2 recruitment by the A_1_AR. **(A)** A_1_AR-NanoBit^®^-βarr2 HEK 293 cells were incubated with or without 1 µM of the positive A_1_AR modulator VCP 171 and stimulated with 0.1 nM, 1 nM, or 100 nM NECA. In combination with 100 nM NECA, VCP 171 led to a significant increase in recruitment when compared to NECA treatment alone. Values are given as means ± SEM (n = 3 independent experiments performed in triplicate). **p < 0.01 value was significantly different (one-way analysis of variance, Dunnett’s *post hoc*). **(B)** A_1_AR-NanoBit^®^-βarr2 HEK 293 cells were incubated with 50 or 100 µM of the GRK2 inhibitor (βARK1) and stimulated with 100 nM or 1 µM NECA. Incubation with the inhibitor together with the higher NECA concentration led to a significant inhibition in recruitment when compared to cells stimulated with 1 µM NECA only. Values are given as means ± SEM (n = 3 independent experiments performed in triplicate). ***p < 0.001 value was significantly different (one-way analysis of variance, Dunnett’s *post-hoc*).

In addition to agonistic and antagonistic effects, modulation of GPCRs can occur. One such modulator of A_1_AR is VCP 171, a 5-substituted 2-aminothiophene. Co-incubation of NanoBit^®^ HEK cells with VCP 171 increased the agonistic effect of NECA (Figure 5A). Under VCP 171 incubation, β-arrestin 2 recruitment increased by 25.5% for 100 nM NECA.

β-arrestin 2 recruitment depends, at least in part, on receptor phosphorylation by GPCR kinases (GRKs). To test whether this dependency can be demonstrated by the A_1_AR NanoBit^®^ assay, the cells were incubated with a specific inhibitor of GRK2 (βARK1 inhibitor). Data presented in Figure 5B demonstrate partial inhibition of β-arrestin 2 recruitment through incubation with the βARK1 inhibitor, which was statistically significant in the case of stimulation with 1 µM NECA.

### Valerian extract Ze 911 induces A_1_AR-mediated β-arrestin 2 recruitment

**FIGURE 6:**
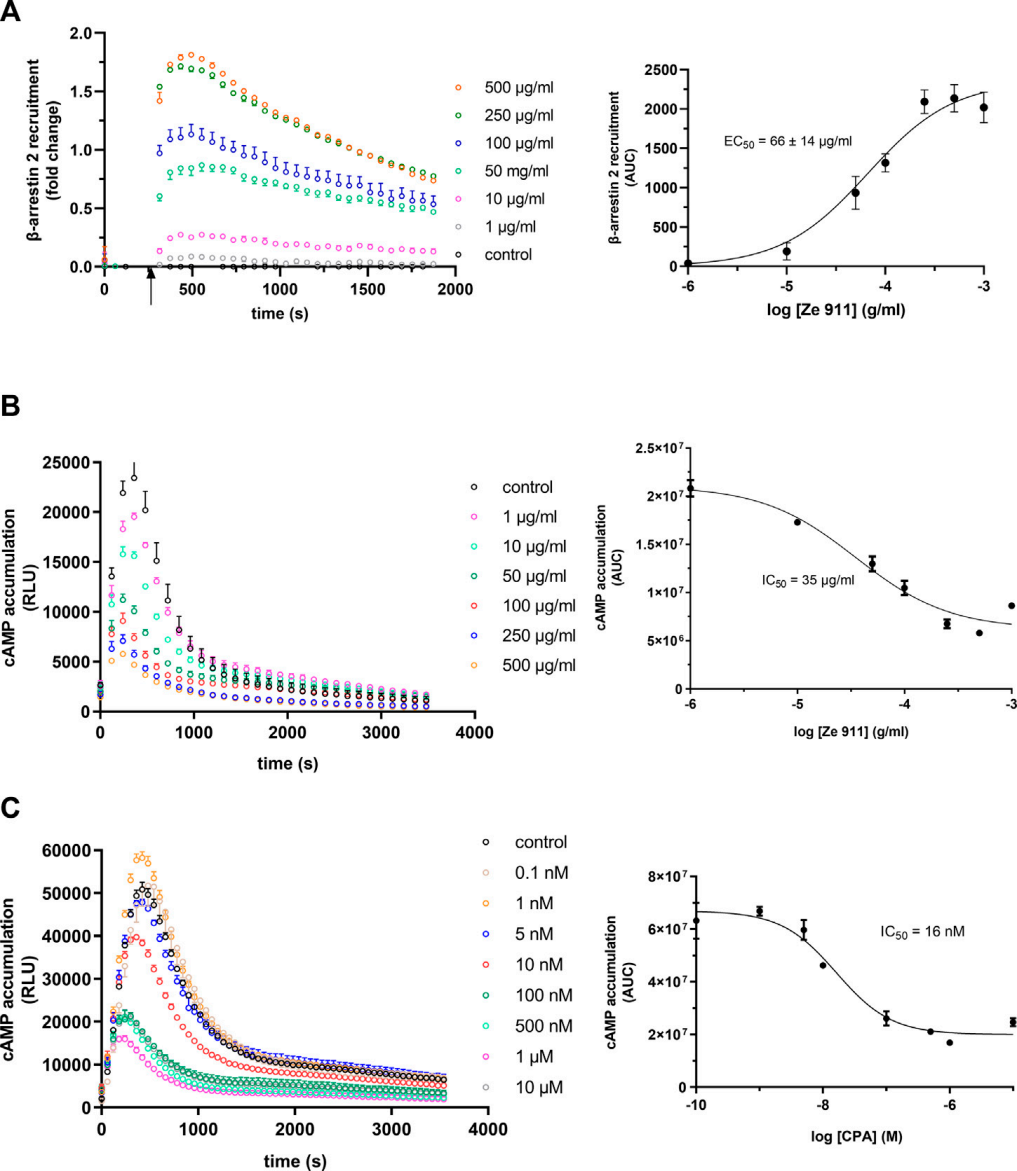
**(A)** Concentration dependency of β-arrestin 2 recruitment in the A_1_AR-NanoBit^®^ reporter assay: At the time point indicated by the arrow, valerian extract Ze 911 was added to A_1_AR-NanoBit^®^-βarr2 HEK 293 cells at the concentrations indicated beside the graph, and luminescence was measured for 30 min. A solvent control of 0.1% DMSO was included. The graph on the left is exemplary for one of three experiments performed in triplicate. The dose–response curve on the right was calculated from all three experiments using areas under the curves (AUCs). Values are given as mean ± SEM (n = 3 independent experiments performed in triplicate). Concentration dependency of Gi activation measured by the inhibition of cAMP production in the GloSensor™ reporter assay for **(B)** valerian extract Ze 911 and **(C)** CPA: HEK GloSensor™ cells were incubated with concentrations indicated, cAMP accumulation was stimulated with 1 µM isoprenaline +1 µM forskolin (timepoint 0), and luminescence was recorded for 60 min. A solvent control of 0.1% DMSO was included. Right: dose–response curves calculated using the area under the curves (AUCs). Values (mean ± SEM) of three wells from one exemplary experiment are shown. Experiments were repeated twice with similar results (see also Supplementary Figure S4A).

A_1_AR plays an important role in the regulation of sleep. In this context, valerian extracts have been demonstrated to show agonistic activity upon A_1_AR, possibly explaining the sleep-inducing effect of this phytopharmaceutical. Until now, no data regarding a possible influence of valerian on the β-arrestin 2 recruitment via A_1_AR exist. As demonstrated by data in Figure 6A, valerian extract Ze 911 induced robust A_1_AR-mediated recruitment of β-arrestin 2. EC_50_ of this activation was calculated to be 66 μg/mL.

Since the literature focused on G_αi_ activation by valerian and not β-arrestin recruitment, a cAMP assay was performed to compare the results (Figure 6B; Supplementary Figure S4). A_1_AR-overexpressing HEK GloSensor™ cells were used to measure the influence of Ze 911 on cAMP accumulation after β-adrenergic stimulation with isoprenaline. Cells were treated with different concentrations of Ze 911. The concentration of cAMP was directly measured after treatment. The cAMP concentration decreased with increasing Ze 911 concentration. The IC_50_ was calculated to be 35 μg/mL. For comparison, cAMP data for CPA were collected in the same manner as for the valerian extract (Figure 6C; for replicates, see Supplementary Figure S4).

## Discussion

Originally identified as adapter proteins mediating receptor desensitization and internalization, β-arrestins are now a recognized component of GPCR signal transduction (Chen and Tesmer, 2022; Jiang et al., 2022). In order to collect sensitive data on the interaction between A_1_AR and β-arrestin 2, an assay based on NanoBit^®^ technology was established and examined in more detail to determine how well it is suited for studying the activation of A_1_AR and subsequent recruitment of β-arrestin 2 (Dijon et al., 2021).

Using the newly established NanoBit^®^ assay, we determined an EC_50_ value of 780 nM for adenosine for A_1_AR-mediated β-arrestin 2 recruitment. In comparison, IC_50_ values for adenosine ranging from 100 to 310 nM were found for adenylate cyclase-mediated cAMP formation (Fredholm et al., 2001; Yan et al., 2003; Müller & Jacobson, 2011). These differences can be explained by differences in the assays themselves. For example, whereas A_1_AR–cAMP assays indirectly measure the inhibitory effect of receptor activation on adenylate cyclase activity stimulated by another substance such as forskolin, the assay presented here directly measures the activity of A_1_AR via immediate β-arrestin 2 recruitment. It has been reported that receptor density affects values of A_1_AR activities obtained by the same agonist (Cordeaux et al., 2000). In addition, concentrations of endogenously produced adenosine or inosine, which may be different in cell cultures and cell membrane preparations, also affect the A_1_AR activity found in the different assays (Cohen et al., 1996; Jarvis and Thompson, 2019).

Nevertheless, the values determined with the different assays are in the same medium-to-high nanomolar range and are, therefore, comparable.

For CPA, Mueller et al. determined an IC_50_ value of 24 nM for inhibition of cAMP production (Müller et al., 2002), which is approximately five times lower than what was found here for β-arrestin 2 recruitment (130 nM). Again, the assays are only partially comparable because cAMP accumulation was measured on isolated membranes of A_1_AR-overexpressing CHO cells treated with adenosine deaminase (ADA), whereas our NanoBit^®^ assay was performed on live HEK cells that were not pretreated with ADA. Therefore, we additionally performed a cAMP assay on live HEK cells (GloSensor™ assay). The IC_50_ value of 16 nM for CPA that we obtained in this assay fits perfectly with the results of Müller et al. (2002). Interestingly, the 5- to 8-fold ratio between cAMP inhibition and β-arrestin 2 recruitment for CPA corresponds to that described previously for adenosine (100–310 nM–780 nM). For the non-specific AR agonist NECA, an EC_50_ value of 121 nM for β-arrestin-2 recruitment was found to be similar to that for CPA. In contrast, the IC_50_ value of 56 nM determined for NECA on A_1_AR by Alnouri et al. for cAMP accumulation is slightly higher than the value determined by the same research group for CPA (Alnouri et al., 2015). This poorer potency of NECA than that of CPA regarding the inhibition of cAMP production via A_1_AR is also confirmed by studies of Cordeaux et al. (2000).

Recruitment of β-arrestins has also become a focus of scientific investigation in recent years because of the tremendous increase in our understanding of the importance of the interplay between the various signaling pathways of GPCRs in the development of diseases, as well as in the efficacy or side effect profile of drugs (Sharma and Parameswaran, 2015; Bagnato and Rosanò, 2019; Bond et al., 2019; Zhai et al., 2022). We have already seen the emergence of the first drugs to exploit of preferred activation of one of several possible signal transduction pathways, also called biased signaling, such as the cardiac drug carvedilol (Wisler et al., 2007; Ibrahim and Kurose, 2012; Kolb et al., 2022; Kenakin 2019). A positive effect of biased agonism on the side effect profile is also thought to be present for A_1_AR (Valant et al., 2014; Baltos et al., 2016; McNeill et al., 2021). For example, benzyloxy-cyclopentyladenosine has been identified as a selective A_1_AR agonist that achieves analgesia without the adverse side effect of cardiorespiratory depression (Wall et al., 2022).

As a non-nucleoside and biased agonist, we chose capadenoson, which was developed as a cardioprotective, highly selective A_1_AR agonist with an improved safety profile (Albrecht-Küpper et al., 2012; Sabbah et al., 2013). To our knowledge, there are no data regarding β-arrestin 2 recruitment mediated by capadenoson-activated A_1_AR. The established NanoBit^®^ assay detected a pronounced partial agonism for this compound, whereas it showed full agonism comparable to that of CPA in the case of adenylate cyclase inhibition (see Supplementary Figure S5). This fits with data collected by Baltos et al., who demonstrated a slight increase in cAMP inhibition as well as a reduction in ERK1/2 and Akt phosphorylation after capadenoson stimulation compared to NECA (Baltos et al., 2016). ERK1/2 phosphorylation in response to A_1_AR activation is at least partially β-arrestin-dependent (Jajoo et al., 2010). Therefore, the reduced ERK1/2 phosphorylation as described by Baltos et al. in the case of capadenoson can be explained by the reduced recruitment of β-arrestin 2 demonstrated in our work.

When calculated via AUCs, A_1_AR-mediated β-arrestin 2 recruitment was approximately two times higher for adenosine, CPA, and NECA than for capadenoson. It should be pointed out that this calculation includes not only the activation of the receptor but also its deactivation/internalization. However, this is also significantly lower in the case of capadenoson due to its reduced activation behavior. To compare different GPCR agonists, it can, therefore, be useful to characterize the activation phase of the receptor only. Hoare and colleagues developed an alternative tool to analyze kinetic signaling data from fluorescent reporter systems ((Hoare et al., 2020a; Hoare et al., 2020b; Hoare et al., 2021) available as plug-ins for Prism). The curves calculated by these plug-ins matched our measurement data very well. Initial rates that reflect the activation kinetics of the different agonists can be calculated from these curves. The difference in the NanoBit^®^ assay calculated on the basis of the initial rates between full agonists such as CPA and the partial agonist capadenoson (up to five-fold) is approximately twice the difference calculated on the basis of AUC (as mentioned previously), more clearly reflecting different receptor activation by the agonists tested here. Calculating the initial rate is, therefore, probably the more sensitive way to investigate different agonists in the case of β-arrestin 2 recruitment measured by the NanoBit^®^ assay.

Assay systems for G protein activation and arrestin recruitment are of interest not only for agonist screening but also for testing receptor modulators that might shift signaling to one pathway or the other. In this context, the results for the allosteric modulator VCP 171 are remarkable because they show that the assay can be readily adapted to determine the activity of modulating agents. The 25% increase in the agonistic effect of NECA fits data reported in the literature (Aurelio et al., 2009; Nguyen et al., 2016). Inhibitory substances for the A_1_AR can also be screened without problems, as shown here exemplarily for DPCPX. The IC_50_ of 105 nM that we calculated fits well with values for DPCPX measured on a model for depression of synaptic transmission mediated by the A_1_AR (Latini et al., 1999).

Internalization of A_1_AR is a slow process compared to that of A_3_AR (Klaasse et al., 2008). It is known that β-arrestin recruitment is regulated in part through receptor phosphorylation by GRKs, but phosphorylation of A_1_AR by GRKs is still controversial (Ramkumar et al., 1993; Nie et al., 1997; Klaasse et al., 2008; Soave et al., 2020). However, in the assay described here, inhibition of GRK2-mediated receptor phosphorylation results in decreased β-arrestin 2 recruitment. The IC_50_ value of 126 µM published by Iino et al. for the βARK-1 inhibitor fits our observations that efficient inhibition of β-arrestin 2 recruitment starts at 50 µM and higher (Iino et al., 2002). Therefore, A_1_AR-mediated β-arrestin 2 recruitment is at least in part regulated by phosphorylation of the receptor through GRK2. The remaining recruitment might be the result of A_1_AR phosphorylation by one of the other ubiquitously expressed GRKs.

After testing chemically defined substances for activating the A_1_AR, we decided to investigate a more complex compound mixture with agonistic activity. Phytopharmaceuticals containing valerian extracts are used as mild sleep-inducing agents (for review, see Borrás et al., 2021; Shinjyo et al., 2020). There is evidence in the literature that this effect is at least partly dependent on A_1_AR activation (Müller et al., 2002; Schumacher et al., 2002; Vissiennon et al., 2006; Lacher et al., 2007). All data on the possible influence of valerian extract or its ingredients on A_1_AR are based on receptor binding data as well as cAMP accumulation. Data on the regulation of β-arrestin 2 recruitment after A_1_AR activation by valerian extracts have not yet been published. The half-maximal inhibition of cAMP accumulation for the valerian extract used in our study was reached at 35 μg/mL in the GloSensor™ assay, whereas it was 900 μg/mL in the activated charcoal absorption assay of Müller et al. (2002). In addition to the differences in the cAMP assays used, the composition of the valerian extract tested by Mueller and colleagues is not directly comparable to that of Ze 911 investigated here, which explains the different IC_50_ values. However, despite all differences, comparable inhibition of cAMP accumulation is described in both publications. Interestingly, β-arrestin 2 recruitment is only two times higher than cAMP inhibition for Ze 911, with an EC_50_ value of 66 μg/mL, and, thus, significantly lower than the 5- to 8-fold ratio described for CPA previously. Ze 911, therefore, appears to have a slight bias toward the recruitment of β-arrestin 2 compared to adenosine or CPA.

The NanoBit^®^ assay for β-arrestin 2 recruitment has been previously reported as a useful tool for other ARs (Storme et al., 2018a; 2018b), but in the case of A_1_AR, it has only been used to monitor the internalization of A_1_AR (Soave et al., 2020), not to investigate the interaction of the receptor with β-arrestins. In the context of biased A_1_AR agonism, one or more downstream signaling pathways such as ERK1/2 activation have often been analyzed instead of direct interaction between β-arrestin and the receptor (Valant et al., 2012; 2014; Baltos et al., 2016). Direct interaction has been studied using the Tango assay (Langemeijer et al., 2013; Laroche and Giguère, 2019), BRET (Navarro et al., 2018; Wall et al., 2022), or techniques such as coimmunoprecipitation or translocation of arrestin-GFP fusion proteins (Ferguson et al., 2002; Tsutsui et al., 2008). Compared to FRET/BRET, the signal-to-noise ratio of NanoBit^®^ assays is very high. For example, signal increases by a factor of 10 can be clearly observed in our assay. This makes the assay extremely sensitive and may allow the establishment of recruitment assays with physiological expression levels of receptors and arrestins in the future. This possibility is also favored by the small size of 19 kDa of the complemented enzyme (Wouters et al., 2018). Initial approaches to introduce the NanoBit^®^ system into cells at the genomic level exist (Oh-hashi et al., 2017; Dale et al., 2019; White et al., 2019).

In conclusion, the presented assay is very well-suited to the study of A_1_AR-mediated recruitment of β-arrestin 2 by different substances. Together with other assays like the cAMP GloSensor™ assay used here and appropriate tools for evaluation, the analysis of biased signaling is also very feasible with this assay.

## Data Availability

The original contributions presented in the study are included in the article/Supplementary Materials; further inquiries can be directed to the corresponding author.

## Supplementary material

SUPPLEMENTARY FIGURE S1 Processing of raw data—A_1_AR NanoBit^®^ reporter assay: At the time point indicated by the arrow, CPA was added to A_1_AR-NanoBit^®^-βarr2 HEK 293 cells at concentrations indicated beside the graph, and luminescence was measured for 30 min. A solvent control of 0.1% DMSO was included. Data for relative luminescence light units (RLU) were plotted as a function of time. To normalize for well-to-well variabilities, each data point was divided by the mean of the first three values using the “remove baseline and column math” function of the software. Next, the solvent control was subtracted using the same functionality of Prism. From this data set, areas under the curves were calculated using the corresponding function and plotted against the concentration of the agonist used (log [agonist] (M)) to receive a dose/response dependency. For each value, RLUs or AUCs (mean ± SEM) of nine wells from three different experiments are shown.

SUPPLEMENTARY FIGURE S2 Results from repeated experiments for concentration dependency of β-arrestin 2 recruitment in the A_1_AR NanoBit^®^ reporter assay: At the time point indicated by the arrow, different agonists were added to A_1_AR-NanoBit^®^-βarr2 HEK 293 cells at concentrations indicated beside the graph, and luminescence was measured for up to 60 min. A solvent control of 0.1% DMSO was included. Values are given as mean ± SEM) (see also Figure 2).

SUPPLEMENTARY FIGURE S3 Results from repeated experiments for β-arrestin 2 recruitment in the A_1_AR NanoBit^®^ reporter assay by different agonists: 10 µM of either CPA, NECA, adenosine, or capadenoson was added to A_1_AR NanoBit^®^-βarr2 HEK 293 cells at the time point indicated by the arrow, and luminescence was measured for 60 min. A solvent control of 0.1% DMSO was included. Values are given as mean ± SEM (see also Figure 3A).

SUPPLEMENTARY FIGURE S4
**(A)** Results from repeated experiments for concentration dependency of β-arrestin 2 recruitment in the A_1_AR NanoBit^®^ reporter assay: At the time point indicated by the arrow, valerian extract Ze 911 was added to A_1_AR NanoBit^®^-βarr2 HEK 293 cells at concentrations indicated beside the graph, and luminescence was measured for 30 min. A solvent control of 0.1% DMSO was included. Values are given as mean ± SEM (see also Figure 6A). **(B)** Dose–response curves from repeated experiments for concentration dependency of Gi activation measured by the inhibition of cAMP production in the GloSensor^TM^ reporter assay for valerian extract Ze 911 (left) and CPA (right): HEK GloSensor^®^ cells were incubated with the concentrations indicated, and cAMP accumulation was stimulated with 1 µM isoprenaline + 1 µM forskolin (time point 0), and luminescence was recorded for 60 min. A solvent control of 0.1% DMSO was included. Values are given as mean ± SEM (see also Figure 6B, C).

SUPPLEMENTARY FIGURE S5
**(A)** A_1_AR agonist-induced reduction in cAMP production measured using the GloSensor^TM^ assay: cAMP accumulation in A_1_AR-GloSensor^TM^ cells was stimulated with 1 µM isoprenaline + 1 µM forskolin, and 10 µM of either NECA, CPA, adenosine, or capadenoson was added at the same time point indicated by the arrow. Luminescence was measured for 60 min. A solvent control of 0.1% DMSO was included. Values are given as means ± SEM. Graphs are exemplary for one of three experiments performed in triplicate. **(B)** A_1_AR agonist-induced reduction in cAMP production is compared to isoprenaline/forskolin-stimulated control and displayed in percent. Values are given as means ± SEM (n = 3 independent experiments performed in triplicate).
