# Supplementary figures and images for: Investigation of adenosine A1 receptor-mediated β-arrestin 2 recruitment using a split-luciferase assay
Source: Front Pharmacol. 2023 May 30;14:1172551. doi: 10.3389/fphar.2023.1172551 (PMC10268005; doi:10.3389/fphar.2023.1172551)

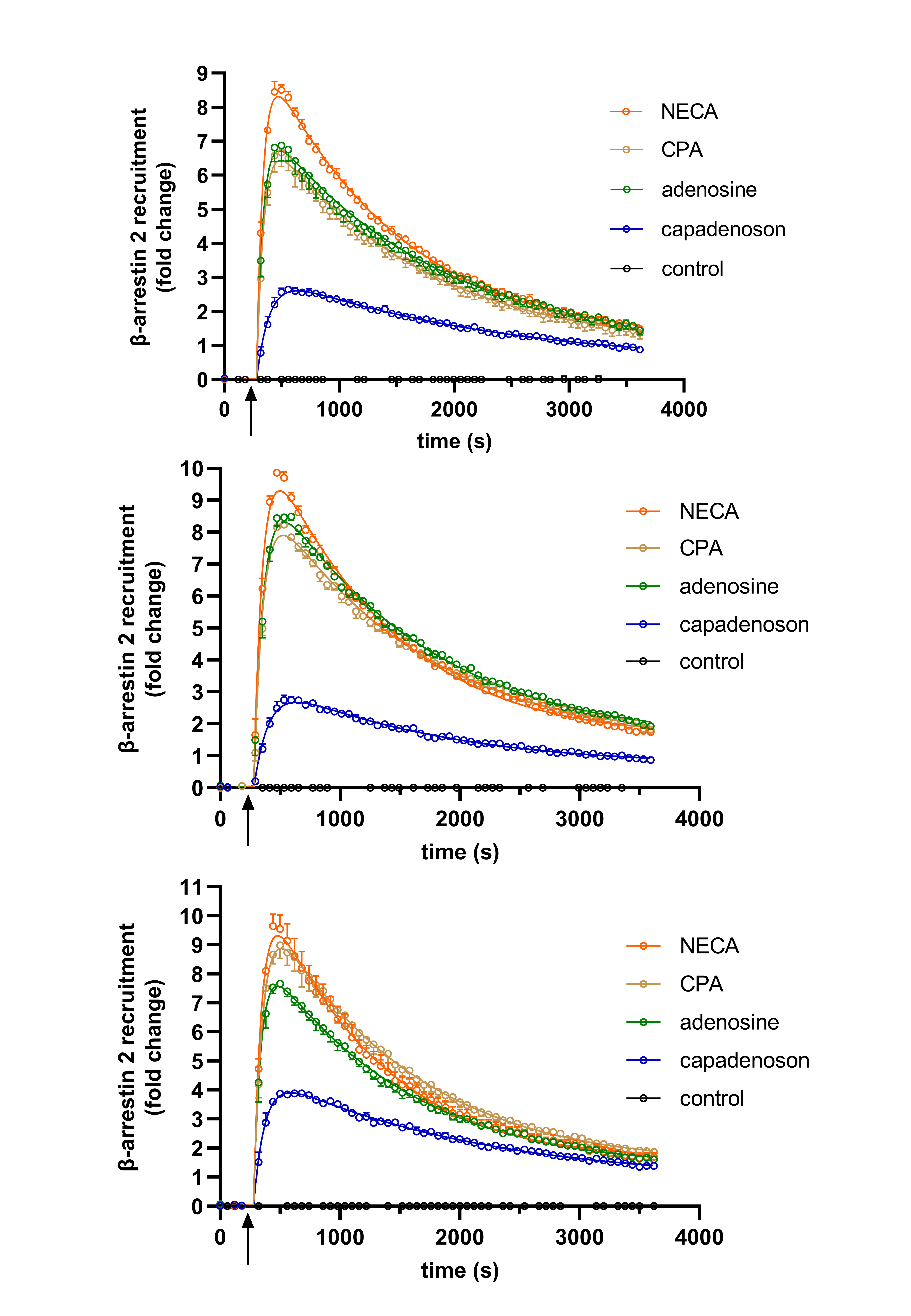

Supplement: Supplementary file 1 [file Image3.tif]

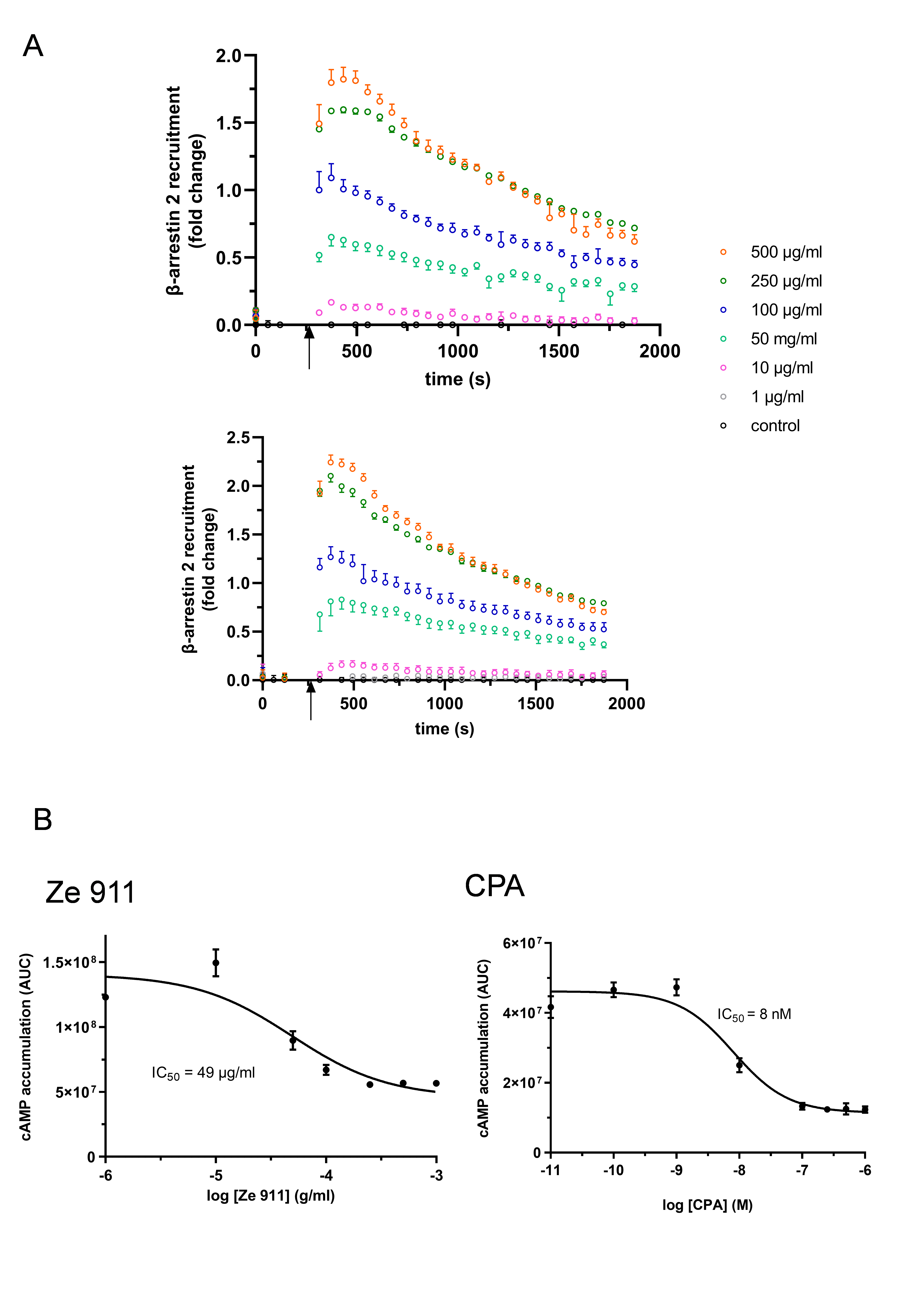

Supplement: Supplementary file 2 [file Image4.tif]

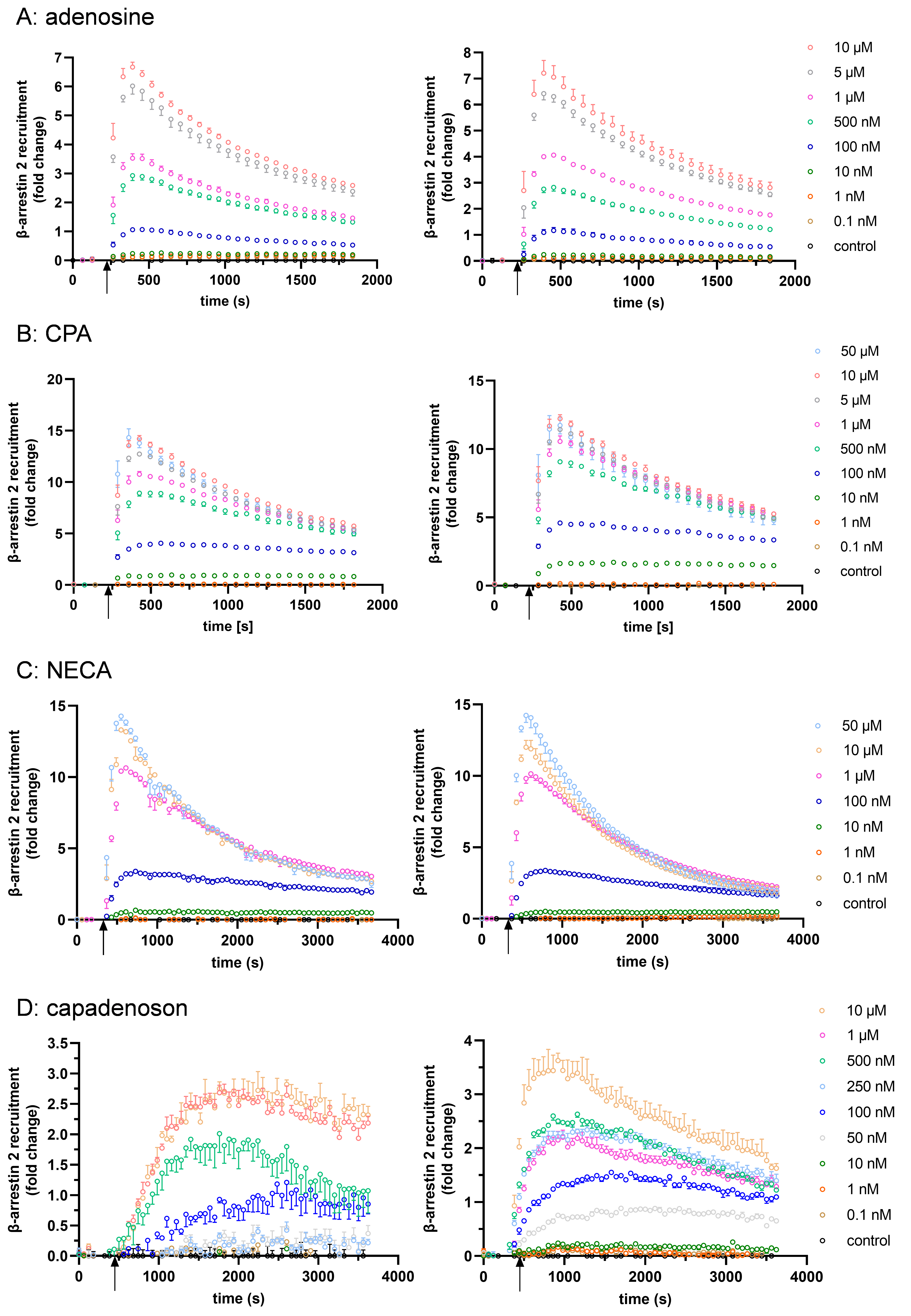

Supplement: Supplementary file 3 [file Image2.tif]

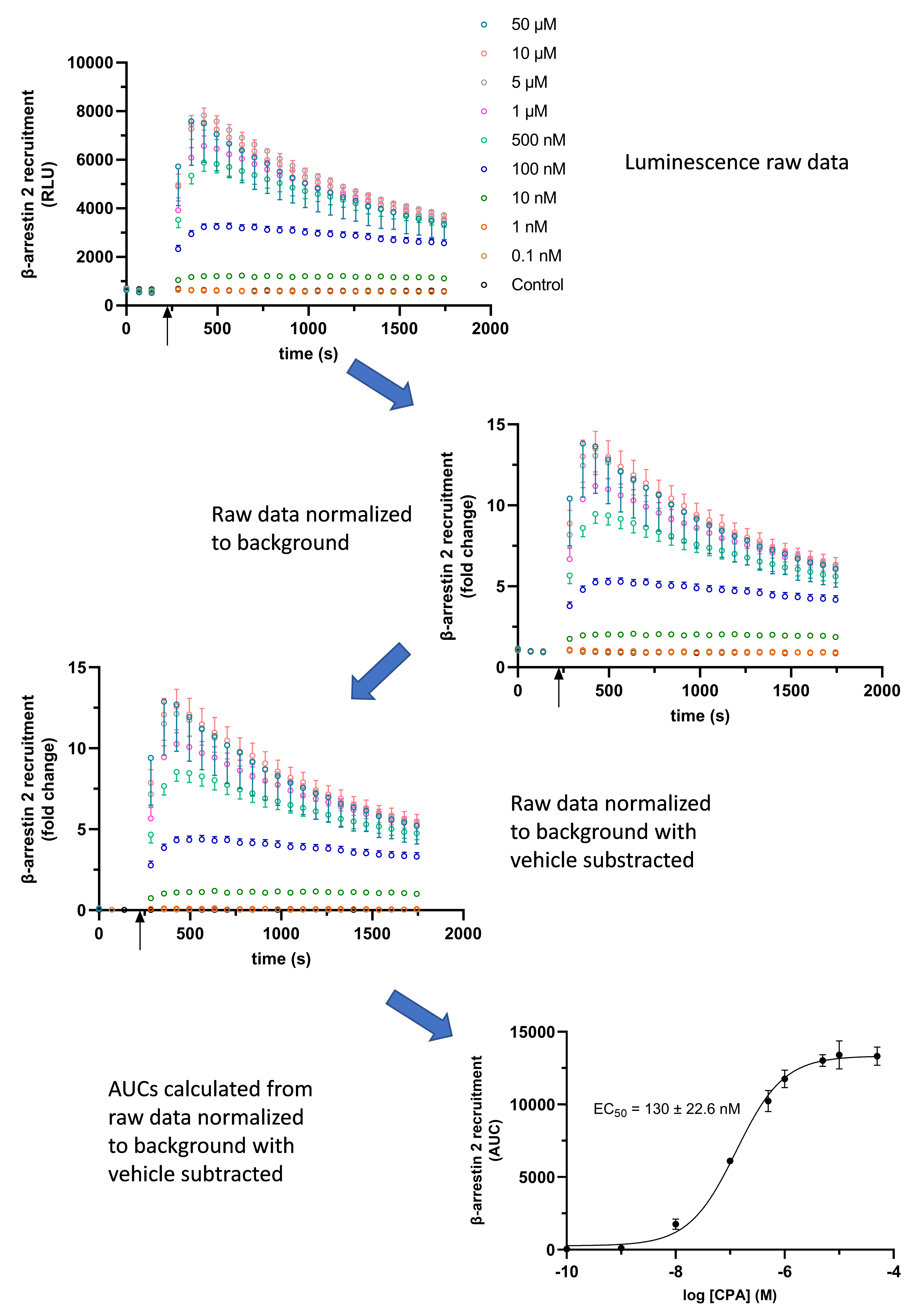

Supplement: Supplementary file 4 [file Image1.tif]

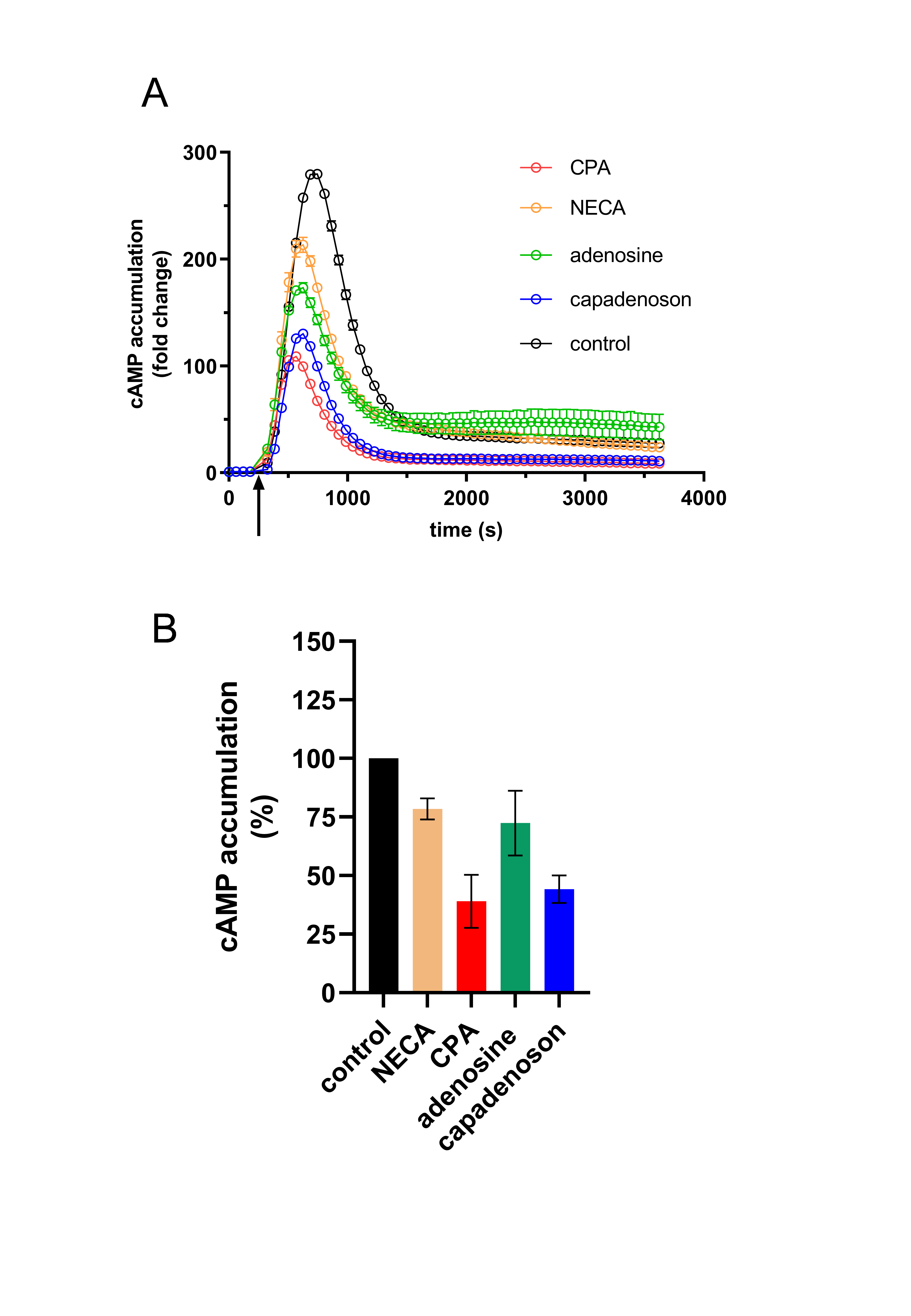

Supplement: Supplementary file 5 [file Image5.tif]
